# Editorial: Artificial Intelligence Applications and Scholarly Publication in Orthopaedic Surgery

**DOI:** 10.1097/CORR.0000000000002658

**Published:** 2023-04-17

**Authors:** Seth S. Leopold, Fares S. Haddad, Linda J. Sandell, Marc Swiontkowski

**Affiliations:** 1 Editor-in-Chief, *Clinical Orthopaedics and Related Research*®, Park Ridge, IL, USA; 2 Editor-in-Chief, *The Bone & Joint Journal*, London, UK; 3 Editor-in-Chief, *Journal of Orthopaedic Research*, St. Louis, MO, USA; 4 Editor-in-Chief, *The Journal of Bone and Joint Surgery*, Needham, MA, USA

Anyone with access to the internet now has free access to artificial intelligence (AI) applications that can quickly develop text-based responses to specific questions. Large language model applications such as ChatGPT have made it possible to write research manuscripts, abstracts, and letters to the editor that are extremely difficult to differentiate from human-derived work (see Appendix; http://links.lww.com/CORR/B99).

This rapid improvement in AI capabilities may offer some benefits to journals, publishers, readers, and, ultimately, patients. For example, large language models such as ChatGPT might—with suitable human oversight—be able to create plain-language summaries of complex research quickly and at scale, which might make the scientific record more accessible to the public [6]. AI-based tools also may facilitate the creation of consistent, clear visual presentations of complex data. And, of course, an exciting feature of transformative technologies is the potential for benefits that we cannot imagine at the outset.

However, misuse of these tools can undermine the integrity of the scholarly record; indeed, there are examples of this happening already. Some even have suggested that large language models should be considered authors. In fact, ChatGPT has been listed as a co-author in published research [4] and even is a registered author in the ORCiD and SCOPUS databases. This practice is inappropriate. Under the authorship guidelines of the International Committee of Medical Journal Editors [3], which all of our journals follow, an author must meet a number of important standards, including being willing to be accountable for all aspects of the work, to ensure that questions related to the accuracy or integrity of the work will be suitably investigated and resolved, to be able to identify which co-authors are responsible for specific parts of the work, and to have confidence in the integrity of the contributions of their co-authors. A large language model has no means to comply with such standards, and, for that reason—as well as, we believe, simple common sense—AI-based tools cannot be authors on scientific papers.

Other important concerns have been raised about the use of AI-driven tools in scientific reporting, including the possibilities that they may produce material that is inaccurate or out of date [2], they may conjure up “sources” that do not exist [1], and—this from the team that built ChatGPT—they may generate “plausible-sounding but incorrect or nonsensical answers,” which the coders have said is “challenging” to fix because “during RL (reinforcement learning) training, there’s currently no source of truth” [5]. We believe that our readers, and the patients for whom they are responsible, deserve better.

For these reasons and others, our editorial boards have agreed on the following standards concerning AI applications that create text, tables, figures, images, computer code, and/or video:

1. AI applications cannot be listed as authors.
2. Whether and how AI applications were used in the research or the reporting of its findings must be described in detail in the Methods section and should be mentioned again in the Acknowledgments section.

Our editorial boards will closely follow the scientific developments in this area and will adjust editorial policy as frequently as required.

## Supplementary Material
