# Supplementary material for: Editorial: Artificial Intelligence Applications and Scholarly Publication in Orthopaedic Surgery
Source: Clin Orthop Relat Res. 2023 Apr 17;481(6):1055–6. doi: 10.1097/CORR.0000000000002658 (PMC10194519; doi:10.1097/CORR.0000000000002658)
Supplement: Supplementary file 1 [file abjs-481-1055-s001.docx]

**Editorial: Artificial Intelligence Applications and Scholarly Publication in Orthopaedic Surgery**

Seth S. Leopold, MD^1^; Fares S. Haddad, FRCS(Orth)^2^; Linda J. Sandell^3^, PhD; Marc Swiontkowski, MD^4^

^1^SS Leopold, Editor-in-Chief, *Clinical Orthopaedics and Related Research^®^*, Park Ridge, IL, USA

^2^FS Haddad, Editor-in-Chief, The Bone & Joint Journal, London, UK

^3^LJ Sandell, Editor-in-Chief, Journal of Orthopaedic Research, St. Louis, MO, USA

^4^M Swiontkowski, Editor-in-Chief, The Journal of Bone and Joint Surgery, Needham, MA, USA

A note from the Editor-in-Chief:

We welcome reader feedback on our editorials as we do on all of our columns and articles; please send your comments to [eic@clinorthop.org](mailto:eic@clinorthop.org).

The authors of this editorial are the Editors-in-Chief of *Clinical Orthopaedics and Related Research®, The Bone & Joint Journal,* the *Journal of Orthopaedic Research,* and *The Journal of Bone & Joint Surgery,* respectively, and this editorial is being published concurrently in all four of those journals. The articles are identical except for minor stylistic and spelling differences in keeping with each journal’s style. Citation from any of the four journals can be used when citing this article.

Each author certifies that there are no funding or commercial associations (consultancies, stock ownership, equity interest, patent/licensing arrangements, etc.) that might pose a conflict of interest in connection with the submitted article related to the author or any immediate family members.

All ICMJE Disclosure of Potential Conflicts of Interest forms for *Clinical Orthopaedics and Related Research®* editors are on file with the publication and can be viewed on request; the Editors’ disclosure statements also appear each month in print on the masthead of *Clinical Orthopaedics and Related Research®.* The ICMJE Disclosure form for the Editor of *The Bone & Joint Journal* is available with the *BJJ* online version of this article. The ICMJE Disclosure form for the Editor of the *Journal of Orthopaedic Research* is available from the Orthopaedic Research Society. The ICMJE Disclosure form for the Editor of *The Journal of Bone & Joint Surgery* is provided with the *JBJS* online version of this article.

This is an open access article distributed under the Creative Commons Attribution License 4.0 (CCBY), which permits unrestricted use, distribution, and reproduction in any medium, provided the original work is properly cited.

S. S. Leopold MD 🖂, *Clinical Orthopaedics and Related Research®*, 300 S. Northwest Highway, Suite 203, Park Ridge, IL, USA, Email: [sleopold@clinorthop.org](mailto:sleopold@clinorthop.org)

Anyone with access to the internet now has free access to artificial intelligence (AI) applications that can quickly develop text-based responses to specific questions. Large language model applications such as [ChatGPT](https://openai.com/blog/chatgpt/) have made it possible to construct research manuscripts, abstracts, and letters to the editor that are extremely difficult to differentiate from human-derived work (see Appendix; http://links.lww.com/CORR/B99).

This rapid improvement in AI capabilities may offer some benefits to journals, publishers, readers, and, ultimately, patients. For example, large language models such as ChatGPT might—with suitable human oversight—be able to create plain-language summaries of complex research quickly and at scale, which might make the scientific record more accessible to the public [6]. AI-based tools also may facilitate the creation of consistent, clear visual presentations of complex data. And, of course, an exciting feature of transformative technologies is the potential for benefits that we cannot imagine at the outset.

However, misuse of these tools can undermine the integrity of the scholarly record; indeed, there are examples of this happening already. Some have suggested that large language models should be considered authors; in fact, ChatGPT has been listed as a co-author in published research [4] and even is a registered author in the ORCiD and SCOPUS databases. This practice is inappropriate. Under the authorship guidelines of the International Committee of Medical Journal Editors [3], which all of our journals follow, an author must meet a number of important standards, including being willing to be accountable for all aspects of the work, to ensure that questions related to the accuracy or integrity of the work will be suitably investigated and resolved, to be able to identify which co-authors are responsible for specific parts of the work, and to have confidence in the integrity of the contributions of their co-authors. A large language model has no means to comply with such standards, and, for that reason—as well as, we believe, simple common sense—AI-based tools cannot be authors on scientific papers.

Other important concerns have been raised about the use of AI-driven tools in scientific reporting, including the possibilities that they may produce material that is inaccurate or out of date [2], they may conjure up “sources” that do not exist [1], and—this from the team that built ChatGPT—they may generate “plausible-sounding but incorrect or nonsensical answers,” which the coders have said is “challenging” to fix because “during RL (reinforcement learning) training, there’s currently no source of truth” [5]. We believe that our readers, and the patients for whom they are responsible, deserve better.

For these reasons and others, our editorial boards have agreed on the following standards concerning AI applications that create text, tables, figures, images, computer code, and/or video:

1. AI applications cannot be listed as authors.
2. Whether and how AI applications were used in the research or the reporting of its findings must be described in detail in the Methods section, and should be mentioned again in the Acknowledgements section.

Our editorial boards will closely follow the scientific developments in this area and will adjust editorial policy as frequently as required.

Seth S. Leopold, MD

Editor-in-Chief, *Clinical Orthopaedics and Related Research®*

Fares S. Haddad, FRCS(Orth)

Editor-in-Chief, *The Bone & Joint Journal*

Linda J. Sandell, PhD

Editor-in-Chief, *Journal of Orthopaedic Research*

Marc Swiontkowski, MD

Editor-in-Chief, *The Journal of Bone and Joint Surgery*

**References**

1. Davis P. Did ChatGPT Just Lie To Me? The Scholarly Kitchen. January 13, 2023. Accessed March 3, 2023. <https://bit.ly/3YkAozF>
2. Flanagin A, Bibbins-Domingo K, Berkwits M, Christiansen SL. Nonhuman "Authors" and Implications for the Integrity of Scientific Publication and Medical Knowledge. *JAMA.* 2023;329:637-639. doi: 10.1001/jama.2023.1344. PMID: 36719674.
3. International Committee of Medical Journal Editors. Defining the role of authors and contributors. Accessed March 3, 2023. <https://bit.ly/3Yf7HmZ>
4. O'Connor S, ChatGPT. Open artificial intelligence platforms in nursing education: Tools for academic progress or abuse? *Nurse Educ Pract.* 2023;66:103537. doi: 10.1016/j.nepr.2022.103537. Epub 2022 Dec 16. Erratum in: *Nurse Educ Pract.* 2023;67:103572. PMID: 36549229.
5. Open AI. Introducing ChatGPT. Accessed March 3, 2023. <https://bit.ly/3SLqgOC>
6. Rosenberg A, Walker J, Griffiths S, Jenkins R. Plain language summaries: enabling increased diversity, equity, inclusion and accessibility in scholarly publishing. Learned Publishing, 2023;36:109-118.
